# Myocardial Infarction and Subdural Hematoma Presenting a Neurosurgical Anesthetic Challenge: Successful Management With Scalp Block and Dexmedetomidine Infusion

**DOI:** 10.7759/cureus.79678

**Published:** 2025-02-26

**Authors:** André Postiga, Mona-Lisa Coutinho, Ricardo Portela e Silva, Carolina Almeida, Francisco Matias

**Affiliations:** 1 Anesthesiology, Unidade Local de Saúde de Coimbra, Coimbra, PRT

**Keywords:** myocardial infarction, neurosurgery, regional anesthesia, scalp block, subdural hematoma

## Abstract

This case report describes the anesthetic management of a 63-year-old patient with acute coronary syndrome (ACS) presenting as non-ST elevation myocardial infarction (NSTEMI) and a concomitant subdural hematoma requiring urgent neurosurgical intervention via burr hole trepanation. Given the high cardiovascular risk associated with general anesthesia in this scenario, the anesthetic team opted for an alternative approach using a scalp block combined with a dexmedetomidine infusion.

The patient was monitored using standard ASA protocols with invasive blood pressure monitoring. Sedation was achieved with dexmedetomidine at 0.7 mcg/kg/hour, and the scalp block was performed using 15 mL of ropivacaine 0.75% and 5 mL of lidocaine 2%. The surgical procedure proceeded uneventfully, with the patient maintaining spontaneous ventilation, hemodynamic stability, and moderate sedation throughout. Postoperatively, the patient was transferred to the coronary care unit for further management, where subsequent coronary angiography revealed three-vessel ischemic heart disease.

Scalp block combined with dexmedetomidine proved to be a safe and effective alternative to general anesthesia, minimizing hemodynamic instability, ensuring adequate analgesia, and avoiding factors that exacerbate myocardial oxygen demand. This approach underscores the importance of multidisciplinary collaboration in complex clinical scenarios and suggests a promising role for regional anesthesia techniques in high-risk neurosurgical patients.

## Introduction

Scalp block is a regional anesthesia technique that aims to block the nerves innervating the scalp. By applying a local anesthetic to specific points where these nerves branch off, it is possible to achieve anesthesia or analgesia of the entire scalp region. This technique was originally introduced over a century ago but has undergone a modern renaissance in intraoperative and postoperative anesthetic management [1].

The scalp block has been increasingly used for effective analgesia, reducing intraoperative and postoperative opioid consumption. In addition to analgesia, it also attenuates autonomic cardiovascular responses to skull fixation, incision, and craniotomy [2]. Due to these benefits, scalp block has become a valuable alternative to general anesthesia, especially in neurosurgery [3].

Subdural hematomas, typically caused by trauma, result from blood accumulating beneath the dura mater, the outermost of the three meninges that surround the brain and spinal cord [4]. Trepanation consists of drilling a hole in the skull using a special instrument called a trepan, which can be used to perform cranial decompression, access brain structures, and implant devices in the brain. Currently, this surgical procedure is the most commonly used method for treating chronic subdural hematoma [5].

Non-ST-segment elevation myocardial infarction (NSTEMI) is diagnosed in patients with symptoms compatible with acute coronary syndrome (ACS) and troponin elevation, but without electrocardiogram (ECG) changes consistent with ST-segment elevation myocardial infarction (STEMI).

The incidence of NSTEMI in recent years has remained unchanged or even increased [6]. Patients with coronary artery disease undergoing non-cardiac surgery are at increased risk of perioperative complications, such as myocardial ischemia, heart failure, arrhythmias, and cardiac arrest, leading to increased morbidity and mortality [7].

The induction of general anesthesia and the start of surgery carry the risk of hypoxia, hypotension, hypertension, decreased hemoglobin, electrolyte abnormalities, arrhythmias, increased inflammation, and acute stress, all of which are factors to avoid in a patient with ACS [8].

This case report describes the use of scalp block and dexmedetomidine as an alternative to general anesthesia in a patient with ACS.

## Case presentation

A 63-year-old man was admitted to the emergency department following an episode of syncope with a fall from his height, resulting in traumatic brain injury. After the fall, he was unable to stand up. Due to living alone, he remained on the floor for approximately 24 hours until his daughter found him. His medical history included hypertension, with no known allergies or relevant family history.

**Figure 1:**
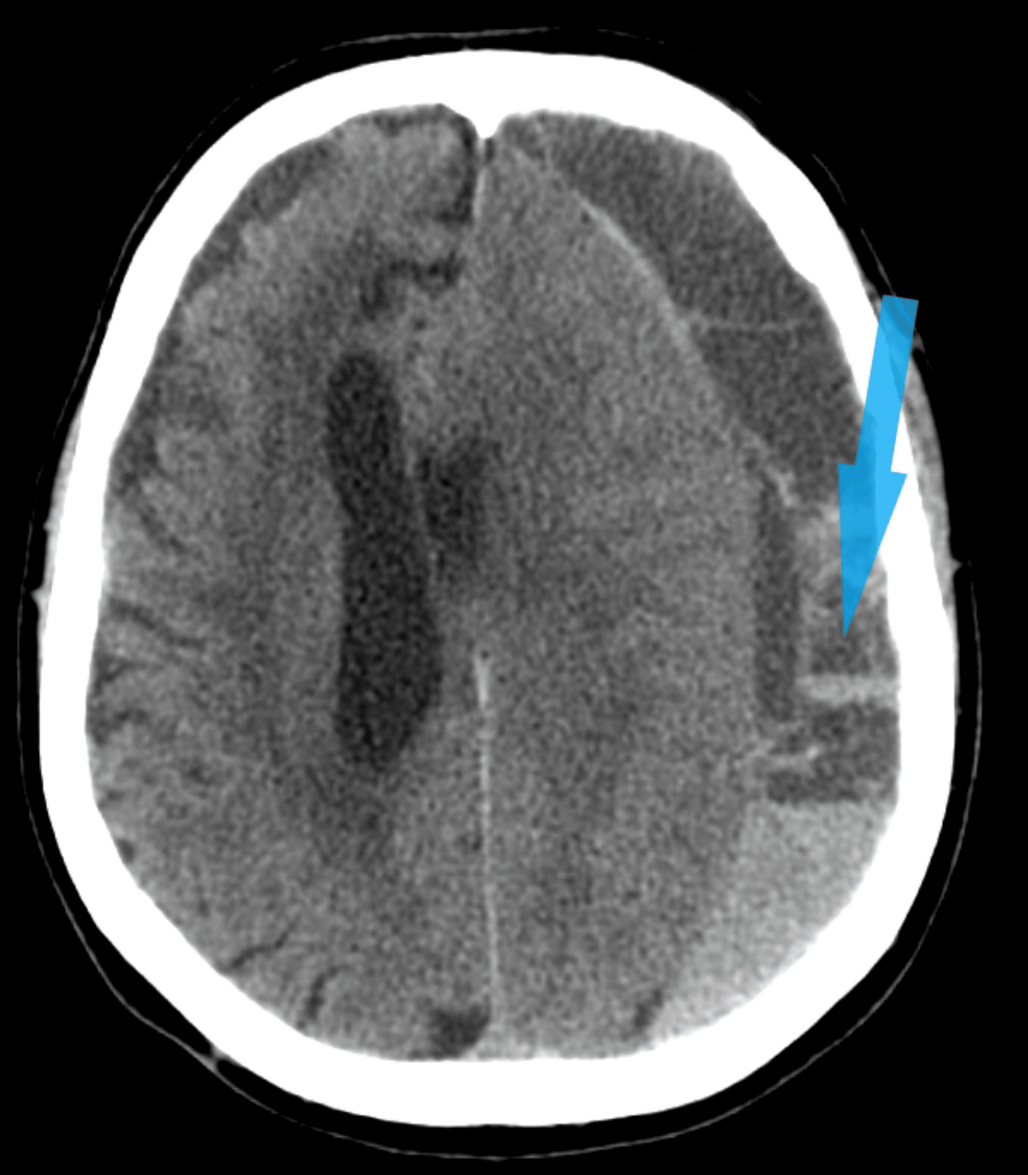
Computerized tomography image: subdural collection with intrinsic gravitational sedimentation, located on the lateral surface of the left cerebral hemisphere; secondary compression of the left cerebral hemisphere, subtotal compression of the left lateral ventricle, obstructive hydrocephalus of the right lateral ventricle.

On arrival at the hospital, he was conscious but disoriented in space and time, with slowed verbalization. His Glasgow Coma Scale (GCS) was 14, and he exhibited grade 4 right hemiparesis (using a scale for grading muscle strength from 0 to 5). A computerized tomography (CT) scan revealed a subdural hematoma requiring surgical intervention (Figure 1).

**Figure 2:**
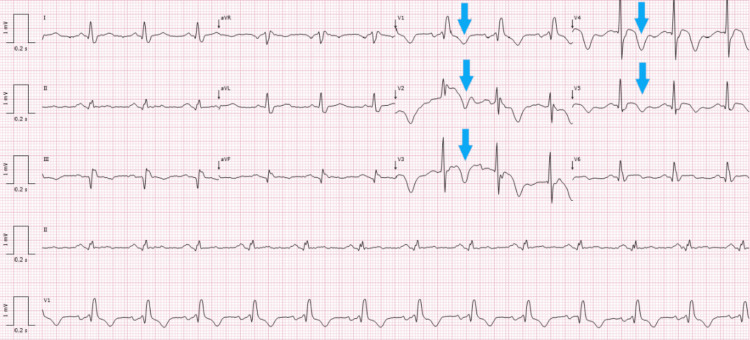
ECG showing T-wave inversion in leads V1-V5. ECG, eletrocardiogram

The patient also reported chest pain associated with the syncope. His ECG showed T-wave inversion in leads V1-V5 (Figure 2), and laboratory analysis revealed elevated high-sensitivity troponin. Cardiology was consulted, and a diagnosis of NSTEMI was made, with an indication for urgent coronary angiography.

Thus, the patient presented with two concurrent emergencies: ACS requiring coronary angiography and a subdural hematoma requiring surgical drainage. A multidisciplinary discussion was held to determine the best course of action. Given the need for dual antiplatelet therapy in the event of stent implantation and the risk of exacerbating the subdural hematoma, the team decided to proceed with hematoma drainage via trepanation.

The team carefully discussed the anesthetic plan and considered the risks and benefits of general anesthesia in the context of the patient's ongoing ACS. A regional anesthesia technique was chosen, involving a scalp block associated with a dexmedetomidine infusion.

Following standard ASA monitoring, an arterial line was placed in the right radial artery for invasive blood pressure monitoring. A dexmedetomidine infusion was initiated at 0.7 mcg/kg/hour. A scalp block was then performed using 15 mL of 0.75% ropivacaine and 5 mL of 2% lidocaine.

With the patient comfortably sedated, spontaneously breathing oxygen via nasal cannula at 2 L/minute, and hemodynamically stable, the surgical procedure began. One hundred micrograms of fentanyl was administered before incision to reinforce analgesia. The subdural hematoma was drained through two trephine holes (frontal and left parietal). The dexmedetomidine infusion was gradually reduced to 0.5 mcg/kg/hour throughout the procedure.

The procedure was uneventful, with the patient maintaining hemodynamic stability and comfort throughout. Following the surgery, he was transferred to the post-anesthesia care unit and subsequently to the coronary intensive care unit. On postoperative day 3, the patient underwent coronary angiography, which revealed three-vessel coronary artery disease.

## Discussion

This case presented the anesthetic team with the difficult challenge of managing a patient with an ongoing ACS requiring urgent neurosurgery. General anesthesia, although widely used in clinical practice, presents significant challenges in patients with cardiac conditions, particularly patients with NSTEMI.

Anesthetic management of patients with ACS should prioritize maintaining myocardial oxygen supply while minimizing oxygen demand [7]. This involves avoiding factors such as pain, tachycardia, and extremes of blood pressure that can exacerbate myocardial ischemia [7,8].

Although there is no clear scientific evidence as to which technique is recommended for patients with ACS, regional anesthesia offers the advantage of avoiding the risks associated with general anesthesia and mechanical ventilation. 

Given the greater risk of hemodynamic instability associated with general anesthesia, we opted for a scalp block combined with a dexmedetomidine infusion. Scalp block has been increasingly used in neurosurgery, particularly for awake craniotomies [9]. Dexmedetomidine, a highly selective α2 receptor agonist, provides anxiolysis, sedation, and analgesia without causing respiratory depression [10].

The combination of scalp block and dexmedetomidine infusion proved to be a satisfactory option, as it allowed a critical neurosurgical procedure to be carried out while maintaining hemodynamic stability and adequate pain control. The patient reported a positive anesthetic experience, denying any pain, anxiety, or stress during the intraoperative period.

The multidisciplinary management of the case with collaboration between the cardiology, neurosurgery, and anesthesiology teams was crucial in ensuring safe and effective treatment of the patient's two urgent conditions: subdural hematoma and NSTEMI. 

Although this case describes the successful use of scalp block and dexmedetomidine infusion in a high-risk patient requiring urgent neurosurgery, further research is needed to consolidate the evidence for this anesthetic strategy and its possible use in elective neurosurgeries, especially in patients with multiple comorbidities.

## Conclusions

The use of a scalp block and dexmedetomidine infusion offers a valuable alternative to general anesthesia for urgent neurosurgical procedures in high-risk patients, namely patients with ACS.

This technique, as demonstrated in this case report, facilitated hemodynamic stability and provided adequate analgesia while minimizing the risks associated with general anesthesia.

Successful management of these complex patients requires a multidisciplinary approach. This combined strategy may contribute to reduced perioperative morbidity and improved clinical outcomes.
